# Dr. Pigot on the Utility of Purging in Typhus

**Published:** 1812-10

**Authors:** J. W. B. Pigot

**Affiliations:** Nicholas-street, Chester


*To the Editors of the Medical and Physical Journal.*

GENTLEMEN,

HOWEVER despicable I may conceive the attack of Medicus to be, and little as any anonymous abuse deserves to be attended to, it seems to me necessary to explain some points where his wilful misrepresentation must have sprung from the most malignant designs. When argument is founded upon data universally allowed, the quarter from whence it proceeds matters little; the motives by which the author is actuated we have no business to inquire into, provided his proofs be satisfactory. But, where an author disputes facts, and accuses another of wilful falsehood, the public has a right to know *who* demands their attention, nor can the public be expected to credit assertions which the author himself is afraid or ashamed to acknowledge, but will require as much evidence to establish belief, as if such anonymous assertions had never appeared.

In the case of Thomas Martin, (published in your Journal for June) which I treated successfully with purgatives in the fever-ward of the Chester Infirmary, I have confined myself to a literal transcript of my clinical notes, which are always deposited at the Infirmary, and open to the inspection of any respectable medical gentleman. A record of cases kept on the plan of the above, must preclude the possibility of misrepresentation. And I consider the register of a public hospital as the *most* satisfactory authority, it being generally witnessed by apprentices and pupils, who are as competent judges of the effects produced by the remedies applied, as the physician himself. I am, perhaps, as anxious to support truth and expose falsehood, as my anonymous critic; though I am by no means conscious any of my remarks have intitled him to use language which neither savours of good sense nor good manners, and he must excuse me if I rather give him credit for private malevolence than public philanthropy. That I have presumed to denominate the fever of Thomas Martin, typhus, seems to be "the very head and front of my offending."

Whilst I have the authority of such men as Currie and Hamilton for my guide, I shall not fear the criticism of Medicus. As it seems probable the works of these two instructive and perspicuous writers have not come within the sphere of his reading, I hope he will be edified by a few extracts from them, and not be Quixote sufficient to accuse them of falsehood and ignorance. The former elegant writer says,* "Since the introduction of scientific arrangements into medicine, diseases have been much reduced in number, and their nature has been more clearly understood. This is especially true of continued fever, which is exhibited by Dr. Cullen under three genera only, synocha, typhus, and synochus; of these genera, however, the synocha, or pure inflammatory fever, without topical inflammation, is confessedly a very rare occurrence in this island; the venerable professor used to declare that he had not met with a single instance of it in forty years' practice. And the typhus and synochus seem to be considered by him as the same disease modified differently, by the difference of climate, season, and constitution. Both are described as contagious, and as occasionally producing each other. Doubtless the typhus, or low contagious fever, is the prevailing fever of this island, and of Europe. It is the epidemic of all our great towns, of our jails, hospitals, and manufactories, its origin and progress are clearly ascertained, and its symptoms generally understood. It is to this fever that the preceding observations chiefly apply." The first case of typhus which Dr. Currie adduces, is that of "a nurse in the fever-ward, who, having several patients under her care, caught the infection. She was seized with violent rigors, wandering pains, succeeded by great heat,

* Ch. viii. V. 1. Currie's Medical Reports, &c.

thirst thirst, and head-ache. Sixteen hours after her first attack, her heat, at the axilla, was 103 Faht., *her pulse* 112 *in the minute, and strong*, thirst great, tongue furred, skin dry.

Dr. Currie adds six other cases of typhus, in all of which the heat of the body was between 101 and 103, and the pulse from 96 to 112.

Thus Medicus may see by the above statement, that *his* opinion, so intemperately advanced, is very far from infallible. We may, however, approve the candid acknowledgment he makes of his ignorance in being unable to detect the presence of fever in the case of Thomas Martin, notwithstanding he had been ill six days, with violent delirium, dry skin, urgent thirst, pungent heat, bowels constipated, with pulse beating 104 in a minute.

I will now take the liberty of stating the opinion of Dr. Hamilton, of Edinburgh, whose practice in the Royal Infirmary of that city, I have attentively witnessed, and been convinced of its efficacy.

"Dr. Cullen admits of two genera of fever only, the intermitting and continued; of the latter typhus or nervous fever, is *most frequent*, and is indeed so general as to be endemial to every country with which we are acquainted. The presence of typhus fever is known by the following symptoms. Some derangement of the stomach, marked by loss of appetite, thirst, sickness, white or loaded tongue, disagreeable taste of the mouth, and most commonly by constipation of the bowels; precede head-ache, languor, debility, and inaptitude for the usual mental and bodily exertions, morbid affections of the surface of the body, of the sanguiferous system, and of different secretions soon succeed; to which, in the more advanced stage, delirium, subsultus tendinum, floccitatio, and singultus, are superadded." The above symptoms follow in succession, and commonly in the order in which I have enumerated them.

"I was appointed physician to the Royal Infirmary upwards of thirty years ago, at this time the cure of typhus was thought to consist chiefly in the removal of atony and spasm of the extreme vessels of the surface of the body. For this purpose, together with other medicines, weak antimonials were given freely, while purgatives were given with extreme diffidence, lest by their operation they should rivet the spasm of the extreme vessels, and increase debility, one of the supposed direct causes of death in fever. These apprehensions may still bias the practice of many, as they certainly did bias mine for a long time."

"I am now thoroughly persuaded that the *full* and regular evacuation of the bowels, relieves the oppression of the

 stomach, stomach, cleans the loaded and parched tongue, and mitigates thirst, restlessness, and heat of surface; and thus the later and more formidable impression on the nervous system is prevented; recovery more certainly and speedily promoted, and the danger of relapsing into fever much diminished."

"My experience in the treatment of typhus enables me to draw the following conclusions.

1st. Purgative medicines are given with safety in typhus, to evacuate the contents of the bowels.

2d. Under this limitation they may and ought to be exhibited at any period from the commencement to the termination of the fever.

3d. Under the same limitation, no circumstance or symptom, in the course of typhus, contra-indicates the exhibition of purgative medicines.

4th. The early exhibition of purgatives relieves the first symptoms, prevents the accession of more formidable ones, and thus cuts short the disease.

5th. In the advanced period of typhus gravior, symptoms that indicate the *greatest danger* were relieved by the evacuation of the bowels, and the patients, in this instance, recovered.

6th. Reconvalescence from typhus, is greatly promoted and confirmed by the preservation of a regular state of the body. The same means secure against the danger of a relapse."

In confirmation of the benefits accruing from the use of purgatives, and in support of the above conclusions, seventeen cases of typhus are added in the appendix, which were almost solely cured by purgative medicines.

Doctor Wilson, in his Essay on the Nature of Fever, p. 147, says, "Every physician must have observed the excellent effects of supporting the due action of the bowels in fever, particularly in its early stages; but no writer has placed this in so clear a point of view as Dr. James Hamilton, of Edinburgh. I have found the most decided advantages from exciting catharsis to the extent he recommends." Dr. W. afterwards says, "I shall only add, that I have very generally seen good effects from spontaneous diarrhœa, even in the worst forms of typhus; an observation which obtruded itself upon me many years ago, when, with other physicians, I was prejudiced against the use of cathartics in this fever, except as far as it was necessary for the regular expulsion of the fœces."*

Dr.

* If Medicus has no particular antipathy to reading, I wish to refer him to some excellent observations of the above author, in further

Dr. Clarke, now of Sidmouth, the well known accurate and scientific reporter of the diseases of Nottingham, has, among others, borne ample testimony to the benefit of the same plan, he says, "the bold plan pursued and recommended by Dr. Hamilton, may excite the astonishment of *a few*, but practice will prove the truth of a system, the result of attentive observation and extensive experience."*

It appears superfluous for me to multiply authorities. Had Medicus possessed a competent knowledge of his profession, or even attentively perused the writings of the authors above mentioned, I am inclined to think his judgment would have been reversed. The next effort of Medicus, is to make a *false* calculation of the medicines given to Hopley, and then call upon the practitioners of Great Britain to join him in thinking such an exhibition impossible.

I am sure no man who was not firmly bent upon distorting facts, for sinister views, and hiding truth, could presume that a patient was disturbed both night and day every half hour, for the space of eight days, for the sake of taking medicine, merely because my record did not directly declare, her nights were not so disturbed. My general order to the nurses is, never to disturb a tranquil and natural slumber for the sake of giving medicine. Thus, I hope, I have amply shewn the futility of every part of the criticism of Medicus. I beg leave to apologize for the length of quotation into which I have been drawn in justification of my practice. For it is not sufficient that a professional man be conscious of his own rectitude, it is also necessary the public do not entertain the least suspicion of it. I am now much more strongly convinced that the favourers of the purgative treatment in fever, and other diseases, ought more generally to make the benefit of their practice known to the world. Their concise descriptions and testimonies, in the different Medical Journals, would reach sequestered places, both in and beyond the British dominions, where the more voluminous work of Dr. Hamilton may not hitherto have travelled.

Since my former communication several cases of typhus

---

further elucidation of this topic, contained in a letter to Dr. Duncan, jun. and published in the Edinburgh Journal for January, 1808, which I hope may lead him to entertain more enlarged and scientific views of fever than he at present seems to possess.

* Vide Ed. Journal, vol. 4th. p. 429.

have have come under my care, which have been happily cured by purgatives. To me, however, it seems more desirable that a *diversity* of practitioners should give *short statements* of their *experience* of these remedies, than that those who have already given their conviction to the public, should multiply the records of their practice.

I have the honor to remain, Gentlemen,

Your most obedient,

J. W. B. PIGOT, M. D.

*Nicholas-street, Chester, August* 1812.

